# A Charrelation Matrix-Based Blind Adaptive Detector for DS-CDMA Systems

**DOI:** 10.3390/s150820152

**Published:** 2015-08-14

**Authors:** Zhongqiang Luo, Lidong Zhu

**Affiliations:** National Key Laboratory of Science and Technology on Communications, University of Electronic Science and Technology of China (UESTC), Chengdu 611731, China; E-Mail: zld@uestc.edu.cn

**Keywords:** blind source separation, DS-CDMA, charrelation matrix, joint diagonalization

## Abstract

In this paper, a blind adaptive detector is proposed for blind separation of user signals and blind estimation of spreading sequences in DS-CDMA systems. The blind separation scheme exploits a charrelation matrix for simple computation and effective extraction of information from observation signal samples. The system model of DS-CDMA signals is modeled as a blind separation framework. The unknown user information and spreading sequence of DS-CDMA systems can be estimated only from the sampled observation signals. Theoretical analysis and simulation results show that the improved performance of the proposed algorithm in comparison with the existing conventional algorithms used in DS-CDMA systems. Especially, the proposed scheme is suitable for when the number of observation samples is less and the signal to noise ratio (SNR) is low.

## 1. Introduction

The problem of blind separation in DS-CDMA systems has attracted extensive attention for the past few years [1,2,3,4,5,6,7,8,9,10,11,12,13,14,15,16,17,18]. Research works related to blind separation for DS-CDMA systems are of particular interest in fields such as anti-jamming in military communications (MILCOM) and satellite communications (SATCOM). There are three main problems which need to be solved, including blind user separation [1,2,3,4,5,6], blind spreading/chip sequence estimation [7,8] and blind interference suppression [9,10,11,12,13,14,15,16,17,18]. The blind separation problems are denominated “blind” to indicate the lack of information concerning the source signals and mixing matrix. The mixing matrix is equivalent to the effect of the channel matrix in DS-CDMA systems. The only prior information utilized is the often soundly justified assumption of statistical independence between the source signals. This technique is called as independent component analysis (ICA), which has important applications for blind separation in wireless communications [1,19,20,21,22,23,24]. So far, some classical ICA algorithms have been proposed to solve blind separation problems in DS-CDMA systems.

A second order blind identification (SOBI) algorithm is used to separate the desired signal and interference signal in spread spectrum communication systems [9]. Fast Independent Component Analysis (FastICA) [2,3,4] and Joint Approximative Diagonalization of Eigen-matrices (JADE) [1,10] are used to separate multiuser signals to resist multiple access interference (MAI) or separate the useful signal and jammer signals for interference mitigation in DS-CDMA systems [10,11,12,13,14,15,16]. JADE and FastICA are classical blind separation algorithms. When evaluating these two algorithms for robustness, separation accuracy and reliability, it is found that the JADE algorithm is robust and reliable, and the FastICA algorithm is not stable and sometimes fails. In general, the existing blind separation techniques utilize the second-order statistics (SOS) and higher-order statistics (HOS) of the observations for source separation [1]. For example, SOBI exploits second order moment information, and FastICA and JADE make use of the four order moments/cumulants information to separate the source signal from mixed signals. Classical HOS are powerful tools in the context of multivariate statistical analysis, often entailing valuable statistical information beyond SOS. However, longer observation intervals might be required in order to fully realize the advantages of HOS over SOS. HOS is better than SOS at the expense of increased computational and notational complexity and compromised statistical stability [1,25].

In this paper, we consider a new generic tool, which offers the structural simplicity and controllable statistical stability of SOS on one hand, yet retains HOS-quality information on the other hand. As is well known, the cumulants are related to higher-order derivatives of the second characteristic function at the origin. However, the new statistical tools are related to lower-order (first and second) derivatives of the second characteristic function away from the origin, at locations called processing-point, and termed charmean and charrelation [25]. The use of charrelation matrices is extremely useful for extracting statistical information in order to establish the optimized cost function for the problem of blind separation work.

Due to the structural simplicity and ample statistical information, we consider using the new statistics instead of the HOS statistics used in blind separation problems for DS-CDMA systems. As far as we know there is little literature reporting on the use of charrelation matrix-based blind separation in DS-CDMA systems [6]. Therefore, the main contribution of this paper is to extend blind separation using charrelation matrix and implement blind user separation and blind spreading/chip sequence estimation. Then the new tools statistic based on off-origin Hessians of the second characteristic function will be discussed and analyzed. We consider a synchronous DS-CDMA systems model. Simulations have been carried out to observe Interference to Signal Ratio (ISR) [24] as a function of sample number of observations and variation in bit error rate (BER) as a function of SNR. A performance comparison of DS-CDMA systems is executed using two assessment criteria to verify the advantages of the proposed method.

The rest of this paper is organized as follows: Section 2 gives a description of the DS-CDMA signal model and describes the relationship between the DS-CDMA signal model and basic blind separation one. The charrelation matrix is illustrated and derived, and the blind user separation and blind spreading/chip sequence estimation are discussed in Section 3. The simulation results and discussions and concluding remarks are given in Section 4 and Section 5, respectively.

## 2. System Model

In this section a concrete discrete-time model of a DS-CDMA system is constructed for the discussion of the problem formulation. The transmitter and receiver structures of DS-CDMA systems involve *K* simultaneous users. We consider the DS-CDMA model as a synchronous one, which is a base band model with fading channel [1,6]. Assuming that the system has *K* users, the signal is sent by user *k* as follows:

(1) $$x_{k}\left(t\right)=\sum_{m=0}^{M-1}b_{k}\left(m\right)c_{k}\left(t-mT\right)$$

which contains the information of *M* symbols $b_{km}$. This symbol $b_{km}$ denotes the *m*th symbol of the *k*th user. $c_{k}\left(\cdot \right)$ is the *k*th use’s binary chip sequence, *i.e.*, the spreading code, supported by [0,T), where *T* is the symbol duration. The signal passed through channel which is assumed to be fixed during one symbol duration:

(2) $$r\left(t\right)=\sum_{m=0}^{M-1}\sum_{k=1}^{K}A_{k}b_{k}\left(m\right)c_{k}\left(t-mT\right)+n\left(t\right)$$

where $A_{k}$ is the channel gain factor of the *k*th user; *M* is the number of symbols per user; *K* is the number of users; n(t) denotes the additive white Gaussian noise with zero mean and variable variance; The chip sequence length (processing gain) is $C=T/T_{c}$, where $T_{c}$ is chip duration. Since the chip sequence $c_{k}\left(t\right)$ is now continuous by definition, it includes not only the binary chips $c_{k}\left(i\right)$, but also a chip waveform p(t). More precisely:

(3) $$c_{k}\left(t\right)=\sum_{i=0}^{C-1}c_{k}\left(i\right)p\left(t-iT_{c}\right)$$

where p(t) is supported by $\left[0,T_{c}\right]$ only. This paper assumes a rectangular waveform for each user. Continuous-to-discrete time conversion of the above model can be realized by a chip-matched filter, which is a simple integrate-and-dump device. Using chip-rate sampling, which means integrating over a chip-duration $T_{c}$:

(4) $$r\left(i\right)=\int_{iT_{c}-T_{c}}^{iT_{c}}p\left(t-\left(i-1\right)T_{c}\right)r\left(t\right)dt$$

The sampled data, which have been obtained by chip-matched filtering using a processing window size of one symbol, can be written as:

(5) $$r\left(m\right)=\sum_{k=1}^{K}A_{k}b_{k}\left(m\right)c_{k}+n\left(m\right)$$

The chip sequence vector $c_{k}$ has a size of C×1, and the noise vector $n_{m}$ has a size of C×1. With a simple manipulation, we can get a compact representation for the data [1,6]:

(6) $$\begin{array}{cc} r(m) & =A_{1}b_{1}\left(m\right)c_{1\left(C\times 1\right)}+\ldots +A_{K}b_{K}\left(m\right)c_{K\left(C\times 1\right)}+n{\left(m\right)}_{\left(C\times 1\right)}\\ = & {\left[c_{1}A_{1},\ldots ,c_{K}A_{K}\right]}_{C\times K}b{\left(m\right)}_{\left(K\times 1\right)}+n{\left(m\right)}_{\left(C\times 1\right)}\\ = & Gb\left(m\right)+n\left(m\right) \end{array}$$

where $c_{k}={\left[c_{1k},c_{2k},\ldots ,c_{Ck}\right]}^{T}$, $n_{m}={\left[n_{1},n_{2},\ldots ,n_{C}\right]}^{T}$, and $b\left(m\right)={\left[b_{1}\left(m\right),b_{2}\left(m\right),\ldots ,b_{K}\left(m\right)\right]}^{T}$. ${\left(\cdot \right)}^{T}$ denotes tranpose. Furthermore, we can obtain:

(7) X=GB+N

where X=[r(1),…,r(M)], B=[b(1),…,b(M)], and N=[n(1),…,n(M)]. This data model Equation (7) is same as the blind source separation one [1,6]. In next section, we will analyze the blind detector related to the model Equations (6) and (7).

## 3. Charrelation Matrix Based Blind Detector for DS-CDMA System

The charrelation matrix can be considered as the off-origin Hessians of the second characteristic function. The theory of charrelation matrix is built on the ideal of “generalized cumulants”, which are defined as the Taylor series coefficients of the second characteristic function same prespecified point in the domain of second characteristic function. 

**Figure 1:**
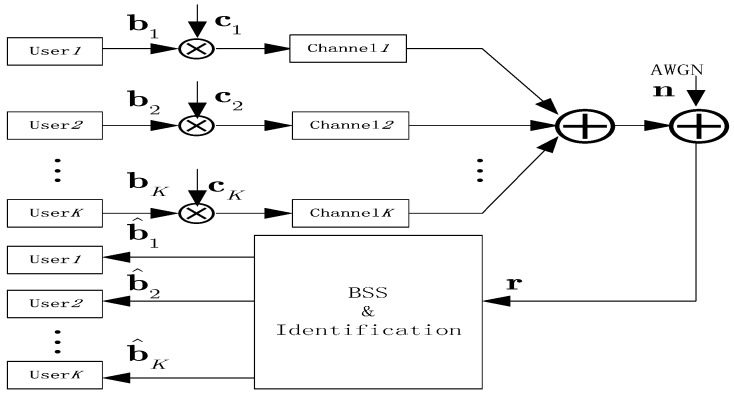
Blind adaptive detector based DS-CDMA Systems.

The prespecified point is processing point, which is away from the zero point. If this point is chosen as the origin, then the generalized cumulants reduce to the traditional cumulants [25]. As an appealing alternative, it is also possible to remain at the more comfortable second-order differentiation, but to move away from the origin. These second order derivatives maintain the convenient form of matrices. The proposed blind adaptive detector for DS-CDMA system is shown in the Figure 1. Next, the charrelation matrix for the blind separation algorithm will be analyzed.

### 3.1. Charrelation Matrix Based Blind Source Separation

Taking into account the blind source separation mixture model linking with DS-CDMA system from above discussion, the system model is rewritten as:

(8) r(m)=Gb(m)+n(m),m=1,…,M

from the perspective of blind separation framwork, the stochastic vetor $r\left(m\right)\in {\mathbb{R}}^{C}$ represents observation signals, and ℝ denotes real field. The components of the stochastic vector $b\left(m\right)\in {\mathbb{R}}^{K}$ correspond to the unobserved source signals. $n\left(m\right)\in {\mathbb{R}}^{C}$ denotes additive Gaussian white noise. The unknown mixing matrix characterizes the way the sources are combined in the observation. The goal of blind separation consists of estimating the mixing matrix $G\in {\mathbb{R}}^{C\times K}$ from the observations and recovering the source signals, on the assumption that the source signal are non-Gaussian and statistically independent. From the single antenna/sensor DS-CDMA reception point of view, in order to have a standard blind separation model available in the receiver, the number of users *K* can be at most *C*, *i.e*., K≤C. That is to say, the mixing matrix **G** is full column rank. Equation (8) is an overdetermined or determined BSS model for K≤C in mixing matrix **G**. In the process of blind separation, the whitening operation is always implemented to convert a model Equation (8) as:

(9) $$\begin{array}{ll} \tilde{r}\left(m\right) & =Qr\left(m\right)\\ & =\tilde{G}b\left(m\right)+\tilde{n}\left(m\right) \end{array}$$

where **Q** is a whitening matrix which is derived in following Section 3.3. After whitening operation, we can obtain that $\tilde{r}\left(m\right)\in {\mathbb{R}}^{K}$, $\tilde{G}\in {\mathbb{R}}^{K\times K}$ and $\tilde{n}\in {\mathbb{R}}^{K}$. Next, the principle of blind separation based on charrelation matrices is illustrated.

Let $u\in {\mathbb{R}}^{K}$ denote an arbitrary (deterministic) vector, called “processing point”. The generalized characteristic function and the generalized second characteristic function of the obesevation vector are defined respectively as:

(10) $$\phi_{\tilde{r}}\left(u\right)≜E\left[\text{exp}\left(u^{T}\tilde{r}\left(m\right)\right)\right]$$

(11) $$\varphi_{\tilde{r}}\left(u\right)≜\log\left(\phi_{\tilde{r}}\left(u\right)\right)$$

E[⋅] denotes the expectation operation. Next we discuss the way to estimate the mixing matrix based on the charrelation matrix of the observations. Replacing $\tilde{r}\left(m\right)$ by its representation and negelecting the noise contribution gets:

(12) $$\phi_{\tilde{r}}\left(u\right)=E\left[\text{exp}\left(u^{T}Gb\left(m\right)\right)\right]=\phi_{b}\left(G^{T}u\right)$$

Furthermore, using the independence property of source vector, the generalized second characteristic function can be rewritten as:

(13) $$\varphi_{\tilde{r}}\left(u\right)=\varphi_{b}\left(G^{T}u\right)=\sum_{i=1}^{K}\varphi_{b_{i}}\left(g_{i}^{T}u\right)$$

where $g_{i}$ is column vector of **G**. As a consequence, deriving the charrelation matrix $\Psi_{\tilde{r}}\left(u\right)$ which is obtained by calculating the second derivative of $\varphi_{\tilde{r}}\left(u\right)$ with respect to **u**, we can obtain the following charrelation matrix $\Psi_{\tilde{r}}\left(u\right)$ (the details are illustrated in Appendix A):

(14) $$\Psi_{\tilde{r}}\left(u\right)=G\Psi_{b}\left(G^{T}u\right)G^{T}$$

with:

(15) $$\Psi_{\tilde{r}}\left(u\right)=\nabla_{u^{T}}\left[\nabla_{u}\varphi_{\tilde{r}}\left(u\right)\right]=\frac{\partial }{\partial u^{T}}\left[\frac{\partial \varphi_{\tilde{r}}\left(u\right)}{\partial u}\right]$$

It is worthwhile to note that $\Psi_{b}\left(G^{T}u\right)$ is a diagonal matrix (the details are illustrated in Appendix B). In a determined blind separation model, source signals are usually separated by multiplying the observations with the pseudo-inverse/inverse of mixing matrix estimate. The estimation of the mixing matrix can be carried out by approximate joint diagonalization (AJD) of a series of charrelation matrices. By choosing a set of processing points $\left\{u_{1},u_{2},\ldots ,u_{L}\right\}$, we can construct a series of charrelation matrices that obey the transformation Equation (14). Then the estimation problem of the mixing matrix can be described as the following joint diagonalization [26,27]:

(16) $${\text{min}}_{G}{\sum_{i=1}^{L}\left\|\Psi_{\tilde{r}}\left(u_{i}\right)-G\Psi_{b}\left(G^{T}u_{i}\right)G^{T}\right\|}_{F}^{2}$$

where ${\left\|\cdot \right\|}_{F}^{2}$ denotes the squared Frobenius norm. In BSS, the diagonal matrices $\Psi_{b}\left(G^{T}u\right)$ contain some statistical or structural properites of sources. The “target matrices” $\Psi_{\tilde{r}}\left(u\right)$ usually denotes estimates of similar matrices pertaining to the observed mixtures. The diagonalization of the matrices $\Psi_{b}\left(G^{T}u\right)$, which is often attributed to the statistical independence of the sources, serves as the key to identifiability of mixing matrix **G** from the matrices $\Psi_{\tilde{r}}\left(u\right)$. The optimization of Equation (16) is a well-known joint diagonalization problem. A number of joint diagonalization methods have been reported, such as in the literatures [26,27]. Among these, a state-of-the-art algorithm called weight exhaustive diagonalization using Gauss iteration (WEDGE) is used to minimize the criterion in Equation (16). The WEDGE approach will not be illustrated in this paper, as details are provided in [26].

### 3.2. Estimation Charrelation Matrix $\Psi_{\tilde{r}}\left(u\right)$

In practical applications the exact charrelation matrix $\Psi_{\tilde{r}}\left(u\right)$ of observations is estimated by sampling of a random variable. The generalized characteristic function of observation vector is estimated as:

(17) $$\phi_{\tilde{r}}\left(u\right)=\frac{1}{M}\sum_{m=1}^{M}\text{exp}\left(u^{T}\tilde{r}\left(m\right)\right)$$

Similarly, the first and second derivatives of the generalized characteristic function is as follows:

(18) $$\Gamma_{\tilde{r}}\left(u\right)≜\frac{\partial \phi_{\tilde{r}}\left(u\right)}{\partial u}=\frac{1}{M}\sum_{m=1}^{M}\text{exp}\left(u^{T}\tilde{r}\left(m\right)\right)\tilde{r}\left(m\right)$$

(19) $$\Xi_{\tilde{r}}\left(u\right)≜\frac{\partial^{2}\phi_{\tilde{r}}\left(u\right)}{\partial u\partial u^{T}}=\frac{1}{M}\sum_{m=1}^{M}\text{exp}\left(u^{T}\tilde{r}\left(m\right)\right)\tilde{r}\left(m\right)\tilde{r}{\left(m\right)}^{T}$$

Based on the previous analysis, the charrelation matrix $\Psi_{\tilde{r}}\left(u\right)$ of the second generalized characteristic function $\varphi_{\tilde{r}}\left(u\right)$ can be easily given by:

(20) $$\Psi_{\tilde{r}}\left(u\right)=\frac{\Xi_{\tilde{r}}\left(u\right)}{\phi_{\tilde{r}}\left(u\right)}-\frac{\Gamma_{\tilde{r}}\left(u\right)\Gamma_{\tilde{r}}^{T}\left(u\right)}{\phi_{\tilde{r}}^{2}\left(u\right)}$$

### 3.3. Blind User Separation and Blind Spreading/Chip Sequence Estimation

Taking into account the problem of blind user separation in a DS-CDMA system, the whitening process will be carried out first in order to simplify the blind separation problem and suppress noise. Using the model Equation (7), the whitening processing is executed as follows:

The autocorrelation of the observations is described as:

(21) $$\begin{array}{ll} R & =E\left[XX^{T}\right]=GE\left[BB^{T}\right]G^{T}+\sigma^{2}I\\ & =U\Sigma U^{T}+\sigma^{2}I\\ & =U\left(\Sigma +\sigma^{2}I\right)U^{T}\\ & =U\Lambda U^{T} \end{array}$$

where $E\left[BB^{T}\right]=\Sigma$, $\Lambda =diag\left(\lambda_{1},\lambda_{2},\ldots ,\lambda_{C}\right)=\mathrm{diag}\left(\sigma_{1}^{2}+\sigma^{2},\sigma_{2}^{2}+\sigma^{2},\ldots ,\sigma_{K}^{2}+\sigma^{2},\sigma^{2},\ldots ,\sigma^{2}\right)$. $\sigma_{i}^{2},i=1,2,\ldots ,K$ are eigenvalues of the signal subspace, which contains the *K* eigenvalues of **R** in descending order. **I** denotes an identity matrix of suitable size. $\sigma^{2}$ is noise variance. The number of active users *K* is known in DS-CDMA systems. The noise variance can be estimated as:

(22) $$\sigma^{2}\approx \left(\lambda_{K+1}+\ldots +\lambda_{C}\right)/\left(C-K\right)$$

The corresponding eigenvalues of signal subspace are $\sigma_{i}^{2}=\lambda_{i}-\sigma^{2},i=1,2,\ldots ,K$. Furthermore:

(23) $$R=\left[\begin{array}{cc} U_{B} & U_{N} \end{array}\right]\left[\begin{array}{cc} \underset{K\times K}{\underset{︸}{\lambda_{B}+\sigma^{2}I}} & 0\\ 0 & \underset{\left(C-K\right)\times \left(C-K\right)}{\underset{︸}{\sigma^{2}I}} \end{array}\right]\left[\begin{array}{c} U_{B}^{T}\\ U_{N}^{T} \end{array}\right]$$

the matrix $U_{B}$ is the size of C×K, which contains the orthonormal signal eigenvectors, and C×(C−K) matrix $U_{N}$ contains the noise eigenvector. $\lambda_{B}=diag\left(\sigma_{1}^{2},\ldots ,\sigma_{K}^{2}\right)$. It is convenient to implement a whitening operation, and **X** can be compressed as $\tilde{X}$:

(24) $$\begin{array}{ll} \tilde{X} & =QX={\lambda_{B}}^{-1/2}U_{B}^{T}\left(GB+N\right)\\ & =\tilde{G}B+\tilde{N} \end{array}$$

The model Equation (24) is a whitened BSS model, $Q={\lambda_{B}}^{-1/2}U_{B}^{T}$ is whitening matrix same as in Equation (9), and $\tilde{G}$ is an orthogonal matrix. Therefore, the problem of Equation (16) is a converted into a orthogonal joint diagonalization to seek a mixing matrix. Assume that the separation matrix is **W** obtained after the optimization of Equation (16). Then the goal of blind user separation is realized by:

(25) $$\hat{B}=sign\left(W\tilde{X}\right)$$

Ideally, there exists the relationship of $W\tilde{G}=I$. In fact, there exists an inherent ambiguity problem in blind source separation. Therefore, $V=W\tilde{G}$ is not an identity matrix but rather a generalized permutation matrix. The ambiguity problem includes amplitude and order ambiguity. The amplitude ambiguity will be eliminated if we assume the covariance of **B** satisfies $E\left[BB^{T}\right]=I$. After the whitening operation, this condition can be obtained easily. The order ambiguity can be solved based on the following principle: 

The recovered source signals can be given by:

(26) $$\hat{B}=W\tilde{G}B=VB$$

where **V** is a generalized permutation matrix, or global matrix, where each column (or row) contains only one non-zero element whose absolute value is 1. It is worthwhile to note that:

(27) $$E\left[\hat{B}B^{T}\right]=VE\left[BB^{T}\right]$$

The global matrix **V** can be estimated by finding the cross-correlation matrix between the vectors of separated signal $\hat{B}$ and source signal **B**. If an estimate of $\tilde{V}$ is acquired, the source signal with proper order can be obtained as:

(28) $$B={\tilde{V}}^{T}\hat{B}$$

In summary, we use the following steps which are shown in Table 1 to overcome the order ambiguity, where we assume each user transmits the short length $M_{p}$ of the pilot symbols.

**Table 1:** Order ambiguity eliminated.

(a) Normalize the pilot symbols so that: $\left(1/M_{p}\right)\sum_{i=1}^{M_{p}}b\left(i\right)b^{T}\left(i\right)=I$ (b) Estimate **V** via the time average as: $\tilde{V}=\left(1/M_{p}\right)\sum_{i=1}^{M_{p}}\hat{b}\left(i\right)b^{T}\left(i\right)$ (c) For each column of $\tilde{G}$, normalized the amplitude of the element which has the maximum absolute-value to be one, and set all of other elements to be zero. Denote the normalized global matrix as $\bar{V}$; (d) Restore the order of the outputs of blind separation by $B={\bar{V}}^{T}\hat{B}$

Next, we will discuss the blind spreading sequence estimate using BSS. According to Equation (7), when the noise **N** is not considered except simulation experiments, we can arrive at:

(29) X=GB

The autocorrelation of **X** is denoted as $R_{X}$, then $R_{X}$ is executed singular value decomposition (SVD), *i.e.*,:

(30) $$R_{X}=UDU^{T}$$

we assume that the $U_{B}\in {\mathbb{R}}^{C\times K}$ denotes the matrix composed of *K* main eigenvectors, then the expanded space of column vector of $U_{B}^{T}\in {\mathbb{R}}^{K\times C}$ belongs to the same space as the expanded space of the column vector of $G^{T}$. Note that there exist a linear transformation relationship between $U_{B}^{T}$ and **G** If the linear transformation is assumed as **A**, we can establish the blind separation model:

(31) $$U_{B}^{T}=AG^{T}$$

The matrix **A** denotes the mixing matix in the BSS model, $G^{T}$ is the source matrix, and the matrix $U_{B}^{T}$ is the observation matrix. Therefore, the blind separation can be used to estimate the matrix $G^{T}$. Assume that **Y** denotes the separated signal. After the separation is executed, the hard decision is carried out for **Y**, namely:

(32) $${\hat{G}}^{T}=sign\left(Y\right)$$

Then we can estimate the spreading sequence from Equation (32) as $\hat{G}={\left[{\tilde{c}}_{1},\ldots ,{\tilde{c}}_{K}\right]}_{C\times K}$. The steps of the proposed method can be outlined as shown in Table 2.

**Table 2:** Blind Adaptive Detector for DS-CDMA system.

(a) The whitening preprocessing of the observation using Equations (21) and (24); (b) Initialize processing point $u_{i},i=1\ldots L$ from the range of [-1, 1]; (c) Estimate charrelation matrices $\Psi_{r}\left(u\right)$ of the whitened signal using Equation (20); (d) Carry out WEDGE joint diagonalization for optimizing problem Equation (16); (e) Seek separation matrix and estimate users’ original signal of DS-CDMA using Equation (25), order ambiguity eliminated by Equations (27) and (28); (f) Based on the model (7), to estimate spreading sequence of DS-CDMA using Equations (30)–(32).

### 3.4. Performance Analysis

In this subsection, we evaluate the blind separation performance of the proposed algorithm compared with the conventional scheme (see details in the simulation analysis) and the HOS-based JADE algorithm. Due to the fact the charrelation matrix incorporates HOS characteristics, which can suppress the Gaussian noise [25]. Therefore, the proposed blind scheme can improve the system performance compared to the conventional scheme in the noise environment. Moreover, the proposed algorithm exploits a “hybrid statistics” (SOS and HOS) manner to extract statistical information, which can acquire more perfect estimated information compared to the JADE with HOS-based manner when the length of signal samples is not enough. In addition, we know that the principle of JADE is joint diagonalization of estimated fourth-order cumulant eigenmatrices [1]. The principle of the proposed method is joint diagonalization of estimated charrelation matrices. From the perspective of computational complexity, the cumulant matrix needs four derivatives of the second characteristic function compared to the two derivatives in charrelation matrix. Finally, the computational complexity of the joint diagonalization method is also considered for both these blind separation algorithms. The computational complexity of the joint diagonalization method of the JADE and the proposed method is $O\left(KM^{4}\right)$ [1] and $O\left(KM^{3}\right)$ [28] respectively. Thus we can know the new method is computed simply and extracts perfect information from samples of observation signals. From the previous analysis, we can evaluate the improved blind separation performance that can be obtained.

In order to carry out more performance assessments, the performance in terms of ISR is displayed in mathematical analysis. The ISR is computed from the estimated mixing matrix by post-multiplying its inverse by the true mixing matrix and averaging the minimum to maximum power ratio in each row of the results. More specifically, if we defined $V=W\tilde{G}$ as the resulting overall “contamination matrix”, $ISR_{ij}≜E\left[V_{ij}^{2}\right]$ is the residual mean contaminating power of source *j* in the reconstruction of source *i*. Thus, in the vicinity of a non-mixing condition $\tilde{G}=I_{K}$, it is easy to observe that $ISR_{ij}\approx E\left[W_{ij}^{2}\right]$. Under the small-errors assumption and sub-Gaussian source signal considered (most communication signals are sub-Gaussian signals), the covariance in the estimation of the elements of $\tilde{G}$, and hence the ISR can be predicted. The ISR performance of JADE can be shown [28] to given by:

(33) $$ISR_{ij}^{JADE}\approx \frac{1}{M}\frac{\kappa_{j}^{4}+\kappa_{i}^{2}+\kappa_{j}^{2}}{\left(\kappa_{j}^{2}+\kappa_{i}^{2}\right)}$$

Likewise, taking into account the diagonal structure (at $\tilde{G}=I$), we can obtain approximatively the ISR performance of the proposed method as follows:

(34) $$ISR_{ij}^{Proposed}\approx \frac{1}{M}\frac{\kappa_{j}^{2}}{\kappa_{j}^{2}+\kappa_{i}^{2}+\kappa_{i}^{2}\kappa_{j}^{2}}$$

where, the $\kappa_{i}$ and $\kappa_{j}$ are statistical moment parameters about unknown ith and jth source. For convenience, without loss of generality, the sources come from the uniform distribution, assuming $\kappa_{i}=\kappa_{j}=\kappa$. We define the ratio of two ISR to evaluate the performance. We can obtain:

(35) $$\eta =ISR_{ij}^{Proposed}/ISR_{ij}^{JADE}=\frac{4\kappa^{6}}{4\kappa^{4}+4\kappa^{6}+\kappa^{8}}=\frac{1}{1+\kappa^{-2}+\kappa^{2}/4}\leq \frac{1}{2}$$

Based on the above analysis, we find that $ISR_{ij}^{Proposed}<ISR_{ij}^{JADE}$ and the ISR performance is inversely proportional to the number of samples *M*. ISR denotes that the ISR is smaller when the **V** matrix is closer to the generalized permutation matrix, and the demixing performance of the algorithm is better. We can know that the proposed method outperforms JADE, and the ISR improvement becomes more pronounced as the number of sample increases. In the next section we will give the simulation results and demonstrate the performance of the DS-CDMA system aided by the proposed method to verify the analyzed case.

## 4. Simulations and Discussions

**Figure 2:**
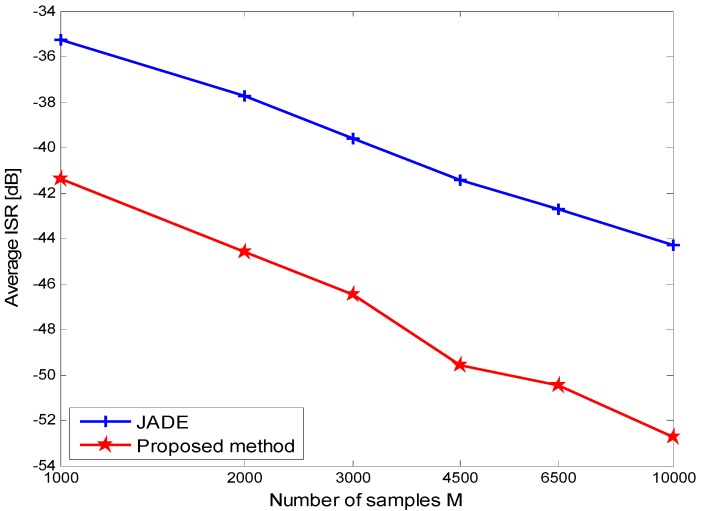
ISR performance comparison of JADE and the proposed method.

**Figure 3:**
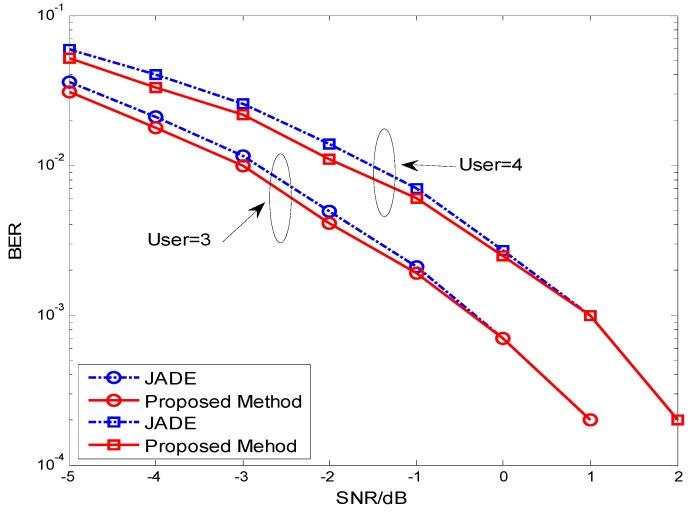
BER performance comparison between JADE and the proposed method for user separation in DS-CDMA systems.

**Figure 4:**
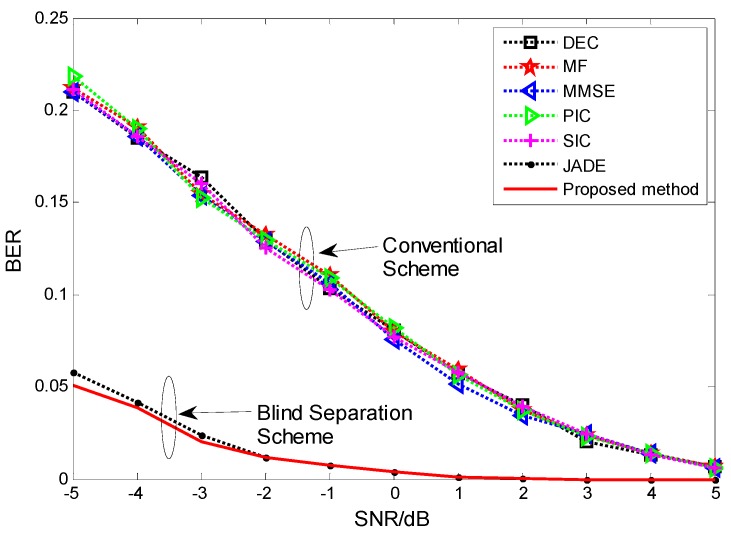
BER performance comparison among the proposed blind separation scheme and conventional scheme.

**Figure 5:**
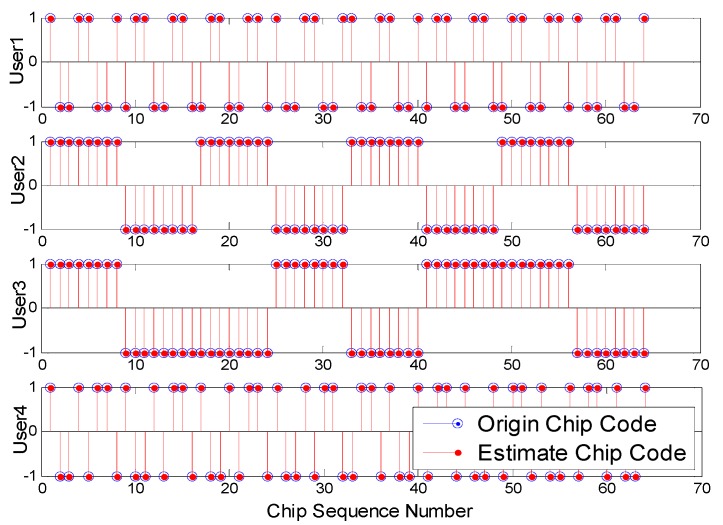
Performance of estimate the chip code for four users in a DS-CDMA system.

**Figure 6:**
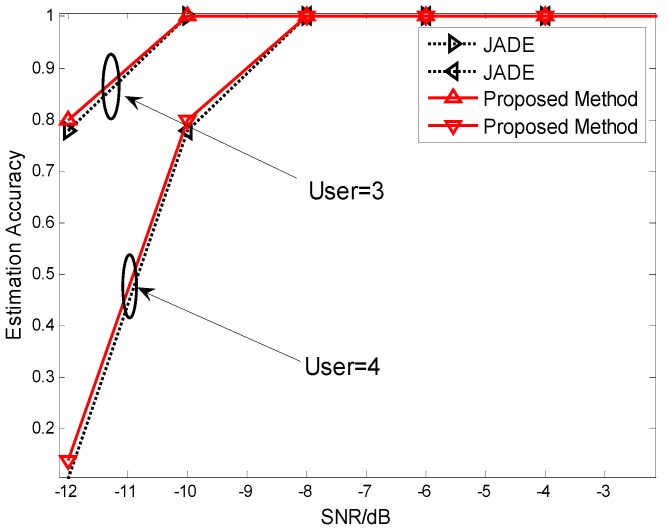
Estimation accuracy of the chip code for different users in a DS-CDMA system with the JADE method and the proposed method.

To demonstrate the effectiveness of our proposed method, simulation experiments are used to illustrate the ISR performance of blind separation by the charrelation matrix and the Bit Error Rate (BER) performance of this method executed in DS-CDMA systems. The results are shown in Figure 2, Figure 3, Figure 4, Figure 5 and Figure 6. The ISR and BER performance index are utilized to show the advantage of performance of the proposed approach.

To begin with, we demonstrate the performance of the ISR, which can be computed in numerical analysis as follows [19]:

(36) $$ISR_{num}=\frac{1}{2K}\left\{\sum_{i=1}^{K}\left[\sum_{j=1}^{K}\frac{{\left|v_{ij}\right|}^{2}}{{\text{max}}_{j}{\left|v_{ij}\right|}^{2}}-1\right]+\sum_{j=1}^{K}\left[\sum_{i=1}^{K}\frac{{\left|v_{ij}\right|}^{2}}{{\text{max}}_{i}{\left|v_{ij}\right|}^{2}}-1\right]\right\}$$

where $V=\left(v_{ij}\right)=W\tilde{G}$ is the unmixing-mixing global matrix, and $W={\tilde{G}}^{-1}$ is the estimated unmixing matrix. 

The simulation parameters are that the number of sources is 4, the mixing matrix is randomly generated, and 10 simulations are implemented, and the channel fading gains of 4 users are set as 1, 0.8, 0.2 and 0.05, which are same as in the following simulation conditions. The other parameters are marked in Figure 2. It is readily seen that the proposed method outperforms JADE from the Figure 2 as previously exposed. Next the Bit Error Rate (BER) of user separation performance in DS-CDMA systems by this method is given. The simulation parameters in DS-CDMA systems are that the number of users is four, the length of spreading code is 31, and Gold Sequence is considered as spreading code, the length of samples is set as short with 1000 bits, 10 simulations are executed, and the modulation mode is BPSK. For comparison, the performance of another BSS algorithm, JADE (using the same data) is indicated as well.

From the simulation results shown in Figure 2 and Figure 3, we can conclude that the new approach has better performance than the JADE algorithms applied in DS-CDMA systems when the number of samples is short and the SNR of receiver signal is low. We also know from Figure 3 that the performance becomes worse with the increasing number of users.

Next, we compared the new method with the conventional scheme and blind scheme used in multiuser detection. The conventional schemes include Decorrelation (DEC), Matched Filter (MF) Minimum Mean-Square-Error (MMSE), Parallel Interference Cancellation (PIC) and Successive Interference Cancellation (SIC). The blind schemes are JADE and the new method. All schemes were tested using Gold code of length C=31. The number of users is K=4. The number of samples is 10,000 bits, the modulation mode is BPSK, and 10 simulations are carried out. The different parameters are marked in Figure 4. According to Figure 4, we can know that blind scheme is superior to the conventional scheme and proposed method is better than the JADE at lower SNR.

In the end, the blind chip sequence estimation is investigated for DS-CDMA systems. The simulation parameters are that the Walsh code of length 64 is tested, and 10 simulations are implemented. 

The different parameters are marked in Figure 5 (SNR = −8 dB) and Figure 6, respectively. Figure 5 and Figure 6 show the chip sequence can be estimated completely at low SNR. From Figure 6, we can know that the proposed method has better performance compared to JADE. In summary, we can acquire the chip sequence with high accuracy in the case of low SNR.

## 5. Conclusions

In this paper, we investigate the charrelation matrix (the generalized covariance matrix) in DS-CDMA systems for blind user separation and blind chip/speading sequence estimation. The unknown mixing matrix is estimated by joint diagonalization of the charrelation matrix of the observations. Theoretical analysis and simulation results show that the proposed blind separation using charrelation matrix performs better than the conventional scheme in low SNR. Especially, the proposed blind separation method has superior performance than that of the existing classical JADE algorithm-based HOS when the number of samplings is short and the SNR of the received signal is low. Furthermore, we can acquire the chip sequence in the case of low SNR and high accuracy assisted blind separation based on the charrelation matrix, so the proposed method has strong ability anti-interference, which is promising in applications for anti-jamming in military communications and satellite communications.

## Appendix

### A. Charrelation Matrix Derivation

Derivation of the charrelation matrix Equation (14) is illustrated in this Appendix. First, the differentiation of formula Equation (13) with respect to **u** gives:

(A1) $$\begin{array}{ll} \frac{\partial \varphi_{r}\left(u\right)}{\partial u} & =\sum_{i=1}^{K}\frac{\varphi_{b_{i}}\left(g_{i}^{T}u\right)}{\partial u}=\sum_{i=1}^{K}\frac{\varphi_{b_{i}}\left(g_{i}^{T}u\right)}{\partial \left(g_{i}^{T}u\right)}\frac{\partial \left(g_{i}^{T}u\right)}{\partial u}\\ & =\sum_{i=1}^{K}\frac{\varphi_{b_{i}}\left(g_{i}^{T}u\right)}{\partial \left(g_{i}^{T}u\right)}g_{i} \end{array}$$

Second, the differentiation of Equation (A1) with respect to $u^{T}$ affords:

(A2) $$\begin{array}{ll} \frac{\partial \varphi_{r}\left(u\right)}{\partial u^{T}\partial u} & =\sum_{i=1}^{K}\frac{\varphi_{b_{i}}\left(g_{i}^{T}u\right)}{\partial {\left(g_{i}^{T}u\right)}^{T}\partial \left(g_{i}^{T}u\right)}g_{i}\frac{\partial {\left(g_{i}^{T}u\right)}^{T}}{\partial u}\\ & =\sum_{i=1}^{K}\frac{\varphi_{b_{i}}\left(g_{i}^{T}u\right)}{\partial {\left(g_{i}^{T}u\right)}^{T}\partial \left(g_{i}^{T}u\right)}g_{i}g_{i}^{T} \end{array}$$

In a simple operation, we can derive a more compact form of the core equation:

(A3) $$\Psi_{r}\left(u\right)=G\Psi_{b}\left(G^{T}u\right)G^{T}$$

### B. Diagonalization Verification

Let $\phi_{b}\left(u\right)$ denote the generalized characteristic function of source signal b(m). Due to the statistical independence of elements of $b\left(m\right)={\left[b_{1}\left(m\right),\ldots ,b_{K}\left(m\right)\right]}^{T}$, we get:

(B1) $$\phi_{b}\left(u\right)=\phi_{b_{1}}\left(u_{1}\right)\cdot \phi_{b_{2}}\left(u_{3}\right)\ldots \phi_{b_{K}}\left(u_{K}\right)$$

where $\phi_{b_{i}}\left(u_{i}\right)=E\left[\text{exp}\left(u_{i}b_{i}\left(m\right)\right)\right],i=1,\ldots K$. Defining $\varphi_{b}\left(u\right)=\log\phi_{b}\left(u\right)$ is called second generalized characteristic function” of source signal b(m). Hence, we obtain:

(B2) $$\varphi_{b}\left(u\right)=\varphi_{b_{1}}\left(u_{1}\right)+\varphi_{b_{2}}\left(u_{2}\right)+\ldots +\varphi_{b_{K}}\left(u_{K}\right)$$

Consequently, the charrelation matrix $\Psi_{b}\left(u\right)$ can be easily obtained:

(B3) $$\begin{array}{ll} \Psi_{b}\left(u\right) & =\nabla_{u}^{T}\left[\nabla_{u}\varphi_{b}\left(u\right)\right]=\frac{\partial }{\partial u^{T}}\left[\frac{\partial \varphi_{b}\left(u\right)}{\partial u}\right]\\ & =diag\left(\frac{\partial^{2}\varphi_{b_{1}}\left(u_{1}\right)}{\partial u_{1}^{2}},\frac{\partial^{2}\varphi_{b_{2}}\left(u_{2}\right)}{\partial u_{2}^{2}},\ldots ,\frac{\partial^{2}\varphi_{b_{K}}\left(u_{K}\right)}{\partial u_{K}^{2}}\right) \end{array}$$
